# Altered domain-specific striatal functional connectivity in patients with Parkinson’s disease and urinary symptoms

**DOI:** 10.1007/s00702-024-02776-0

**Published:** 2024-04-25

**Authors:** Noemi Piramide, Rosa De Micco, Federica Di Nardo, Giuseppina Caiazzo, Mattia Siciliano, Mario Cirillo, Antonio Russo, Gioacchino Tedeschi, Fabrizio Esposito, Alessandro Tessitore

**Affiliations:** 1 Department of Advanced Medical and Surgical Sciences, University of Campania “Luigi Vanvitelli”, Napoli, Italy; 2 Neuropsychology Laboratory, Department of Psychology, University of Campania “Luigi Vanvitelli”, Caserta, Italy

**Keywords:** Parkinson’s disease, Drug-naïve, Urinary symptoms, Functional connectivity, MRI

## Abstract

**Background:**

In this study, we aimed at investigating the possible association of urinary symptoms with whole-brain MRI resting-state functional connectivity (FC) alterations from distinct striatal subregions in a large cohort of early PD patients.

**Methods:**

Seventy-nine drug-naive PD patients (45 PD-urinary^+^/34 PD-urinary^−^) and 38 healthy controls (HCs) were consecutively enrolled. Presence/absence of urinary symptoms were assessed by means of the Nonmotor Symptom Scale - domain 7. Using an a priori connectivity-based domain-specific parcellation, we defined three ROIs (per each hemisphere) for different striatal functional subregions (sensorimotor, limbic and cognitive) from which seed-based FC voxel-wise analyses were conducted over the whole brain.

**Results:**

Compared to PD-urinary^−^, PD-urinary^+^ patients showed increased FC between striatal regions and motor and premotor/supplementary motor areas as well as insula/anterior dorsolateral PFC. Compared to HC, PD-urinary^+^ patients presented decreased FC between striatal regions and parietal, insular and cingulate cortices.

**Conclusions:**

Our findings revealed a specific pattern of striatal FC alteration in PD patients with urinary symptoms, potentially associated to altered stimuli perception and sensorimotor integration even in the early stages. These results may potentially help clinicians to design more effective and tailored rehabilitation and neuromodulation protocols for PD patients.

**Supplementary Information:**

The online version contains supplementary material available at 10.1007/s00702-024-02776-0.

## Introduction

Urinary symptoms are frequent in Parkinson’s disease (PD), with patients experiencing both storage and voiding difficulties, such as urgency, incontinence, polyuria, nicturia and voiding hesitancy (Chaudhuri and Schapira 2009; Picillo et al. 2017). Moreover, these nonmotor symptoms have been associated with increased risk of falls, admission to long-term care and overall worse motor and cognitive outcomes in PD patients. (Chaudhuri and Schapira 2009; Picillo et al. 2017)

In healthy subjects, the neural control of the micturition cycle has been shown to involve different brainstem and spinal cord regions. Indeed, in normal condition, during the storage phase the bladder fills, and ascending signals of fullness/emptiness are directed toward the pontine micturition center and the periaqueductal gray within the midbrain.(Fowler et al. 2008; Griffiths 2015; de Groat et al. 2015) Parallelly, descending outputs from the brainstem are directed to the spinal cord and eventually to the pelvic floor muscles. After the storage phase, the voiding reflex is triggered by the bladder. The voiding stimulus may be voluntarily delayed by the activation of the urethral sphincter and the pelvic floor muscles leading to the inhibition of the ascending signals to the brain. Alternatively, the storage phase switches to the micturition phase. (Fowler et al. 2008; Griffiths 2015; de Groat et al. 2015) Inhibitory afferent signals from the brain to the spinal cord centers determine the relaxation of the urethral sphincter and pelvic floor muscles, while the bladder contracts. (Fowler et al. 2008; Griffiths 2015; de Groat et al. 2015)

Furthermore, a few forebrain regions have been proposed to be actively involved in the regulation of this complex function. Indeed, previous neuroimaging studies have shown that the basal ganglia, the insula, the anterior cingulate (ACC) and prefrontal (PFC) cortices are differently implicated and proposed to work in three parallel urinary-related neural circuits. (Fowler et al. 2008; Griffiths 2015; Kitta et al. 2015; de Groat et al. 2015)

The first circuit, encompassing mainly the insula and anterior PFC, is responsible for visceral and homeostatic sensation. (Griffiths 2015; de Groat et al. 2015) It has also a crucial role for decision-making upon urinary stimuli in social context. (Griffiths 2015; de Groat et al. 2015) In healthy brains, during the storage phase, the insula is activated, and the anterior PFC is deactivated, leading to the suppression of voiding. (Griffiths 2015; de Groat et al. 2015)

The second circuit consists of the dorsal ACC and the supplementary motor area (SMA), and it is considered as a back-up continence mechanism. (Griffiths 2015; de Groat et al. 2015) The dorsal ACC registers and elaborates emotional responses to stimuli, and detects the interoceptive bladder sensation, while the SMA is involved in the planning and organization of motor outputs related to voiding. (Griffiths 2015; de Groat et al. 2015) During bladder filling, this circuit is mainly inactivated. (Griffiths 2015; de Groat et al. 2015)

Finally, the third circuit encompasses mainly temporal areas and basal ganglia, and it is potentially involved in the subconscious monitoring of bladder events. (Griffiths 2015; de Groat et al. 2015) It has been suggested that this circuit may be relevant to ensure that the voiding is safe, by involuntary retrieving and recognizing the social context from episodic memory. (Griffiths 2015; de Groat et al. 2015)

Several lines of evidence suggest that these areas are also direct targets of PD-related neurodegenerative processes, (Krack et al. 2010) and are engaged in multiple parallel re-entering circuits emerging from different subregions of the striatum, that is: (i) the “motor loop”, connecting the SMA with the putamen, (ii) the “cognitive loop” linking the dorsal caudate with the dorsolateral PFC, (iii) and the “limbic loop” establishing crucial connections between the orbitofrontal cortex and the ventral caudate and between the ACC and the nucleus accumbens.

Taking into account these considerations, we hypothesize that a characteristic path of degeneration within the cortico-striatal loops may parallel with the involvement of overlapping urinary-related neural circuits in PD patients. Thus, in this study we investigated the pattern of whole-brain striatal functional connectivity (FC) as emerging from distinct striatal seed regions in a cohort of drug-naïve PD patients with and without urinary symptoms. To this aim, we divided the striatum into three subregions (per each hemisphere) according to cortical-striatal anatomical connections, namely the sensorimotor, cognitive and limbic striatum, and then applied a seed-based FC analysis approach to compare the two PD subgroups as well as patients and healthy controls. We hypothesize that the presence of urinary symptoms may parallel with specific FC rearrangements occurring over the three urinary-related neural circuits in PD patients even in the early stages.

## Methods

### Study population

The study sample was recruited from an ongoing project enrolling consecutive patients with early PD diagnosed according to the modified diagnostic criteria of the UK Parkinson’s Disease Society Brain Bank at the Movement Disorders Unit of the First Division of Neurology at the University of Campania “Luigi Vanvitelli” (Naples, Italy). In this study, drug-naïve PD patients with a modified Hoehn and Yahr (mH&Y) stage ≤ 2.5 were included. Exclusion criteria were: (1) PD onset before age 40 years; (2) any previous treatment with dopaminergic, anticholinergic, antidepressant, or other centrally acting drugs, to rule out a potential effect on functional connectivity from these agents; (3) relevant cognitive impairment; (4) indwelling catheter, renal dialysis, cardiac failure requiring diuretics, urinary tract infection, prostate or bladder cancer, uncontrolled bladder outlet obstruction, pelvic organ prolapse, and previous urogynecologic surgery; and (5) any other clinically significant medical condition.

Presence/absence of urinary symptoms were assessed by means of the Nonmotor Symptom Scale (NMSS) - domain 7. (Chaudhuri and Schapira 2009) Patients were considered to present urinary symptoms (PD-urinary^+^) if they scored ≥ 1. Consequently, patients with NMSS - domain 7 = 0 were labeled as PD-urinary^−^.

Thirty-eight healthy age and sex-matched controls (HCs) with no family history of PD or parkinsonism, and no history of urologic disorders or urinary symptoms were also included in the study. History of any neurological symptoms as well as medical and surgical significant comorbidities were also considered as exclusion criteria for controls.

All the subjects signed their written informed consent. The study was approved by the ethics committee of the University of Campania “Luigi Vanvitelli”, Naples, Italy.

### Clinical evaluation - motor and nonmotor symptoms

Disease severity and motor performance were assessed using the mH&Y stages(Hoehn and Yahr 1967) and the Unified Parkinson’s Disease Rating Scale (UPDRS) part III (UPDRS-III)(Goetz et al. 2008). The presence and severity of other nonmotor symptoms were assessed by means of the NMSS.

### Clinical evaluation – neuropsychological and behavioral symptoms

All patients underwent an extensive neurological and neuropsychological assessments as previously reported (De Micco et al. 2021b; Micco et al. 2021a) (see Supplementary information for more details).

### Statistical analysis of clinical data

Demographic data between PD subgroups and HCs were compared using ANOVA models. T-tests were used to compare motor, nonmotor, neuropsychological, and behavioral variables between PD-urinary^+^ and PD-urinary^−^. Chi-square was used to assess differences in the distribution of categorical variables. Cohen’s d was used to explore the effect size for T-test. Analyses were all Bonferroni-corrected for multiple comparisons. Analyses were performed with SPSS version 23 (SPSS Inc., Chicago, IL, USA).

### Imaging parameters

Magnetic resonance images were acquired on a General Electric 3 Tesla MRI scanner equipped with an 8-channel parallel head coil. fMRI data consisted of 240 volumes of a repeated gradient-echo echo planar imaging T2*-weighted sequence (TR = 1508 ms, axial slices = 29, matrix = 64 × 64, field of view = 256 mm, thickness = 4 mm, interslice gap = 0 mm). During the functional scan, subjects were asked to simply stay motionless, awake, with their eyes closed. Three-dimensional high-resolution T1-weighted sagittal images (GE sequence IR-FSPGR, TR = 6988 ms, TI = 1100 ms, TE = 3.9 ms, flip angle = 10, voxel size = 1 × 1 × 1.2 mm3) were acquired for registration and normalization of the functional images as well as for voxel-based morphometry (VBM) analysis.

### FMRI preprocessing

*f*MRI data preprocessing was performed in Matlab® (The MathWorks, Inc., www.mathworks.com) with the toolbox Data Processing Assistant for Resting-State fMRI (DPARSF, Yan and Zang 2010, http://rfmri.org/DPARSF), which is based on Statistical Parametric Mapping (SPM, http://www.fil.ion.ucl.ac.uk/spm) and Data Processing & Analysis of Brain Imaging (DPABI, Yan et al. 2016, http://rfmri.org/DPABI). Each individual rs-fMRI time series was first corrected for the different slice scan acquisition times by specifying the number of slices, the slice acquisition order and the reference slice. The alignment of the first volume of each subject time-series to the corresponding anatomical 3D-T1w image was obtained via affine transformation; then, all T1w images from all subjects were normalized to the MNI space with the non-linear diffeomorphic DARTEL approach; e14last, the coregistered functional data were normalized to the MNI space with the transformations obtained during the DARTEL procedure and functional scans were resampled to 3 × 3 × 3 mm voxel sizes.

To reduce the residual effects of head motion, as well as the effects of respiratory and cardiac signals, second-order motion and physiological nuisance correction were performed using a linear regression approach: the regression model included 24 motion-related predictors (Friston et al. 1996), with 6 head motion parameter time-series, their first-order derivatives and the 12 corresponding squared parameter time-series; the mean time-courses from a white matter mask and a cerebrospinal fluid mask (as obtained from 3D-T1w spatial segmentation) were added as two additional predictors. In order to account for residual motion-related spikes, an additional spike-related regressor was created from the framewise displacement time-series, i.e. a predictor with a value of 1 at the time points of each detected spike and a value of 0 elsewhere (Lemieux et al. 2007; Satterthwaite et al. 2013). Finally, the image time series were band-pass filtered between 0.01 Hz and 0.5 Hz and spatially smoothed with an isotropic 6-mm full width at half maximum (FWHM) Gaussian kernel.

To minimize the potential effects of head motion and possibly exclude subjects exhibiting excessive amounts of motion, we applied severe inclusion criteria: the six estimated head motion parameters (3 translation and 3 rotation) were considered and subjects exhibiting head translations > 3 mm and/or head rotations > 3 degrees were excluded from consecutive analyses. Then, the mean framewise displacement value (FD) was estimated as an additional measure of total instantaneous head motion (Power et al. 2012) and the percentage of spike-corrupted volumes in each time-series was calculated. Potential spike-corrupted volumes were identified where the FD value exceeded a threshold of 0.5 mm; at this stage, subjects for whom the percentage of corrupted volumes exceeded 50% in the scan were also excluded from the analyses.

Rs-fMRI time series were imported in BrainVoyager and transformed to the Talairach space for seed-based analysis.

### Seed-based connectivity analysis

A seed-based analysis was performed to study FC from striatal loop areas, including bilateral limbic, cognitive, and sensorimotor regions, which were defined according to previous works (Biondetti et al. 2021) to the entire brain. Six ROIs were defined from the anatomical brain atlas “striatum-con-label-thr50-3sub-1 mm” which is freely available for download from the FSL website (see https://fsl.fmrib.ox.ac.uk/fsl/fslwiki/Atlases/striatumconn for all details). Two versions of the atlas exist: the first version comprises of 3 subdivisions (limbic, executive, and sensorimotor), whereas the second comprises of 7 subdivisions (limbic, executive, rostral-motor, caudal-motor, parietal, occipital, and temporal). More specifically we used the probabilistic connectivity striatal atlas with subdivision into three subregions according to cortical-striatal anatomical connections from right and left putamen, right and left caudate and right and left ventral striatum, resulting in six ROIs in total that were used as seed for the FC analysis.

To calculate the functional connectivity maps corresponding to each selected ROI, the mean regional time course was extracted from all ROI voxels and this was correlated against the time-courses from all voxel of the brain. Separate correlation (r) maps were produced for each subject of each group and ROI. Fisher’s transform z = 0.5 Ln [(1 + r)/(1– r)] was applied to these correlation maps before entering a second-level random-effects statistical analysis where the main and differential effects for the two studied groups were summarized as t-statistic maps. For these computations, we used an in-house written Matlab script which was based on an open source Matlab tool called Neuroelf which is freely available at https://neuroelf.org. This tool provides an easy and convenient interface from NIFTI data processed in any software to the commercial software BrainVoyager (Brain Innovation B. V., Maastricht, The Netherlands, www.brainvoyager.com) that we used here to perform the second-level voxel-based analysis and ultimately overlay the resulting statistical contrast (t-statistics) maps onto an high resolution standard T1 template image for display at the voxel- and cluster-level statistical thresholds as resulting from the correction for multiple comparisons. This analysis was carried out by treating the individual subject map values as random observations at each voxel, thereby the classical analysis of variance (ANOVA) was performed at each voxel to map the whole-brain distribution of the seed-based functional connectivity for the difference among groups. From this model, for each seed, statistical contrasts were derived for the following comparisons: PD-all vs. HC, PD-urinary^+^ vs. HCs, PD-urinary^−^ vs. HCs, PD-urinary^+^ vs. PD-urinary^−^. An inclusive mask was created from a standard T1 template image, to define the search volume for multiple comparisons correction: this mask included the entire brain after excluding the striatal loop ROIs that were used as seed for the analysis. To correct for multiple comparisons in the voxel-based analysis, regional effects resulting from the voxel-based comparative tests in the search volume, were only accepted for compact clusters surviving the joint application of a voxel- and cluster-level threshold chosen with a nonparametric randomization approach. Namely, an initial voxel-level threshold was set to *p* = 0.001 (uncorrected) and a minimum cluster size was estimated after 1000 Monte Carlo simulations that protected against false positive cluster up to 5% (*p* = 0.05, cluster-level corrected) (Forman et al. 1995; Eklund et al. 2016; Anderkova et al. 2017).

Individual FC z-scores for both the patients’ subgroups were extracted from regions identified in the above analyses. A univariate analysis of variance was performed between the individual FC z-scores in PD-urinary^+^ and PD-urinary^−^, running NMSS items scores and use of medications for urinary disturbances as covariates.

### Partial correlation analysis

Partial correlation coefficients were computed between imaging (i.e., FC z-scores) and clinical data (i.e., NMSS - domain 7 scores) in PD-urinary^+^. Analyses were adjusted for age and sex. A *p* < 0.05 was considered statistically significant. Analyses were performed with SPSS version 23 (SPSS Inc. Chicago, IL).

*VBM analysis* (see Supplementary information).

## Results

### Clinical results

Forty-five PD urinary^+^ and 34 PD urinary^−^ patients were enrolled in the study. No demographic differences were detected between PD patients and HCs.

PD-urinary^+^ and PD-urinary^−^ patients were comparable in terms of demographic and clinical variables, except for the presence of urinary and gastrointestinal symptoms (Table 1).

**Table 1:** Demographical and clinical data of PD-urinary^+^ and PD-urinary^−^ patients

		HCs (38) mean ± SD	PD-urinary^+^ (45) mean ± SD	PD-urinary^−^ (34) mean ± SD	F-test	P-value	Cohen’s d
	*Demographic variables*						
Sex (M/F)		18/20	26/19	20/14	0.605	0.541	
Age (y)		61.3 ± 4.7	62.7 ± 7.7	59.9 ± 9.8	1.532	0.220	
Education (y)		10.7 ± 3.3	11.4 ± 4.7	11.0 ± 4.5	0.026	0.744	
Disease duration (m)		-	15.4 ± 7.6	14.8 ± 9.5	1.807	0.725	
	*Motor variables*						
mH&Y		-	1.6 ± 0.5	1.4 ± 0.5	0.835	0.081	
UPDRS III		-	19.8 ± 8.1	18.5 ± 7.8	1.071	0.473	
Most affected side (R/L)		-	21/24	17/17	0.394	0.769	
	*Cognitive variables*						
MoCA		-	22.0 ± 3.0	22.5 ± 3.6	0.712	0.531	
Z-score executive		-	-0.7 ± 1.2	-0.5 ± 1.0	0.701	0.471	
Z-score visuospatial		-	-1.1 ± 1.2	-1.6 ± 1.2	0.355	0.191	
Z-score attention/WM		-	-0.5 ± 1.1	-0.3 ± 0.8	0.070	0.437	
Z-score language		-	0.0 ± 1.0	-0.0 ± 0.9	0.124	0.988	
Z-score memory		-	-0.9 ± 0.6	-0.8 ± 0.9	1.663	0.916	
MMSE		28.5 ± 1.1	-	-	-	-	-
	*Nonmotor variables*						
NMSS-1, Cardiovascular			0.7 ± 1.4	0.1 ± 0.4	11.261	0.025	
NMSS-2, Sleep/fatigue			4.3 ± 4.3	1.9 ± 3.0	3.763	0.008	
NMSS-3, Mood/apathy			6.7 ± 7.7	4.1 ± 4.8	1.701	0.093	
NMSS-4, Perceptual problems			0.0 ± 0.2	0.0 ± 0.0	6.782	0.218	
NMSS-5, Attention/Memory			2.2 ± 4.2	0.8 ± 2.0	2.492	0.063	
NMSS-6, Gastrointestinal			2.9 ± 2.4	0.9 ± 1.5	12.818	*< 0.001*	0.99
NMSS-7, Urinary		-	4.0 ± 3.9	0.0 ± 0.0	22.940	*< 0.001*	1.45
NMSS-8, Sexual function			0.6 ± 2.0	0.5 ± 1.6	0.394	0.716	
NMSS-9, Miscellaneus			3.3 ± 2.7	1.9 ± 3.1	0.080	0.042	
	*Behavioral variables*						
Beck depression inventory		-	9.9 ± 8.9	6.5 ± 5.5	7.386	0.065	
Parkinson Anxiety Scale		-	10.2 ± 7.8	7.0 ± 5.5	1.440	0.158	
Parkinson Fatigue Severity Scale		-	2.3 ± 1.0	1.8 ± 0.7	3.171	0.053	
Apathy Evaluation Scale		-	32.5 ± 7.5	32.0 ± 6.8	0.093	0.813	

P-values refer to ANOVA models, pairwise T-test or χ2 as appropriate. A *P* < 0.002 (0.05/25) was considered statistically significant. Cohen’s d refers to the effect size of T-test [(Cohen’s d range of values: small (d = 0.2), medium (d = 0.5), and large (d = 0.8)]. *Abbreviations*: HCs, healthy controls; PD, Parkinson’s disease; y, years; m, months; mH&Y, modified Hoehn & Yahr Scale; R, right; L, left; NMSS, Nonmotor Symptoms Scale; MMSE, Mini-Mental State Examination; MoCA, Montreal Cognitive Assessment; SD, standard deviation; UPDRS, Unified Parkinson’s Disease Rating Scale; WM, Working memory

Among the PD-urinary^+^ patients, 13 (all males) were treated with medications for urinary disturbances (5 with tamsulosin, 3 with dutasteride, 5 with finasteride). PD-urinary^−^ patients were not taking any medications that are used to improve urinary symptoms (i.e. alpha blockers, anticholinergic, 5-alpha reductase inhibitors, phosphodiesterase-5 inhibitors).

### ROI-based functional connectivity results

All PD patients vs. controls (Figure S1; Table 2).

Compared to HCs, PD patients showed decreased FC between the right sensorimotor ROI and the bilateral substantia nigra and between the right sensorimotor ROI and the right fusiform gyrus. Compared to HCs, PD patients showed decreased FC between both the right and left limbic ROIs and the dorsal ACC, and between the right limbic ROI and left anterior insula and between the right limbic ROI and the right anterior dorsolateral PFC. Compared to HCs, PD patients showed decreased FC between the right cognitive ROI and the left angular gyrus, and increased FC between the right cognitive ROI and the left posterior insula.

**Fig. 1:**
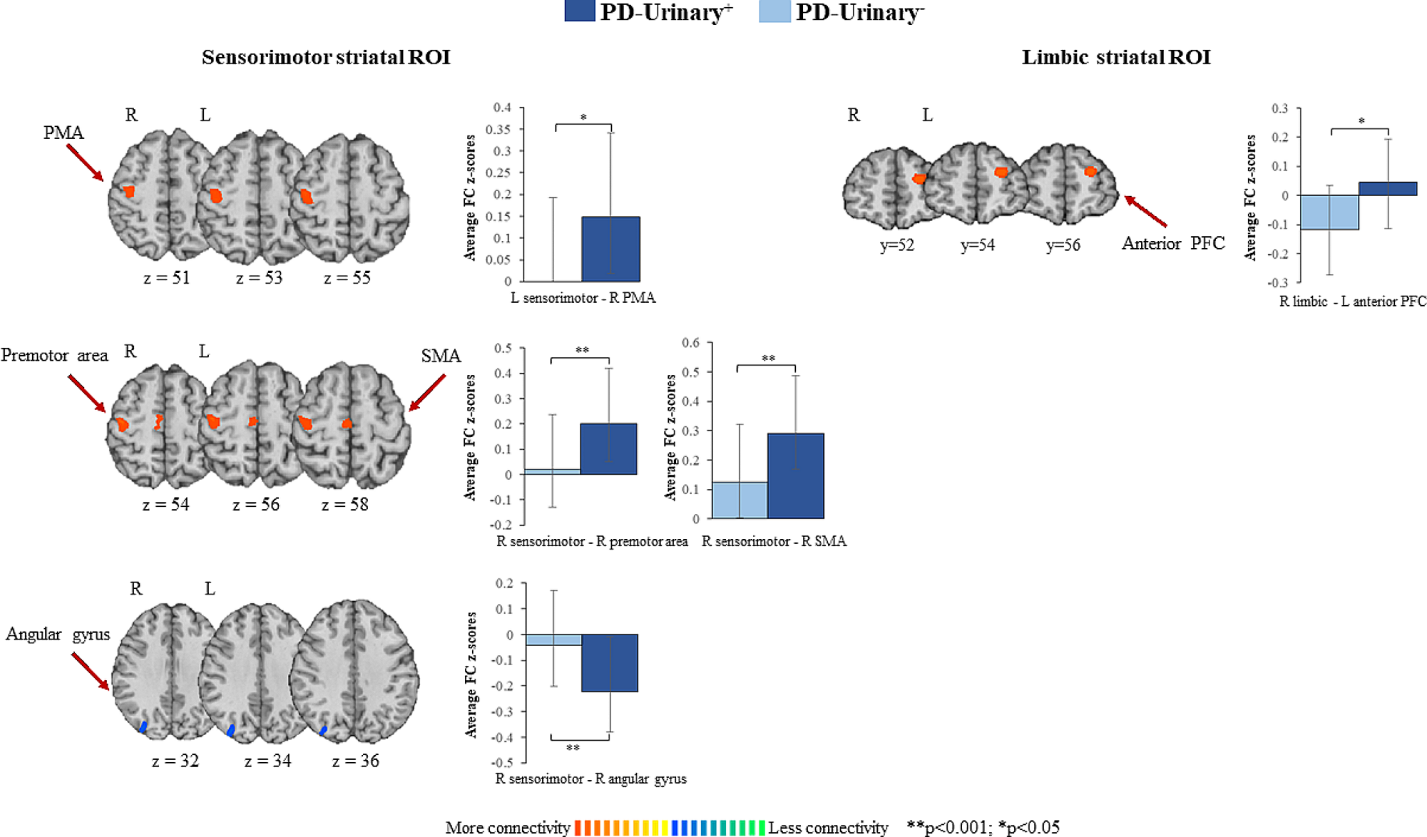
Striatal connectivity changes differentiating PD-urinary^+^ from PD-urinary^−^. Seed-based significant connectivity differences between PD-urinary^+^ and PD-urinary^−^ and bar graphs of the average functional connectivity z-scores (for bilateral results, the mean of the two areas has been reported; please refer to Table 2 for full comparisons). Cold colors represent less, and hot colors represent more connectivity in PD-urinary^+^ (*p* < 0.05 cluster-level corrected). Abbreviations: HCs, healthy controls; PD, Parkinson’s disease; PFC: prefrontal cortex; PMA: primary motor area; ROI: region of interest; STG: superior temporal gyrus

PD-urinary^+^ vs. PD-urinary^−^ (Fig. 1; Table 2).

Compared to PD-urinary^−^, PD-urinary^*+*^ patients showed increased FC between the both the right and the left sensorimotor ROIs and the right premotor/SMA and primary motor area (PMA), and decreased FC between the right sensorimotor ROI and right angular gyrus. PD-urinary^+^ patients showed increased FC between the right limbic ROI and left anterior PFC relative to PD-urinary^−^ patients. These results remain significant even after controlling for gastrointestinal symptoms together with all other NMSS items scores (Table S1).

**Fig. 2:**
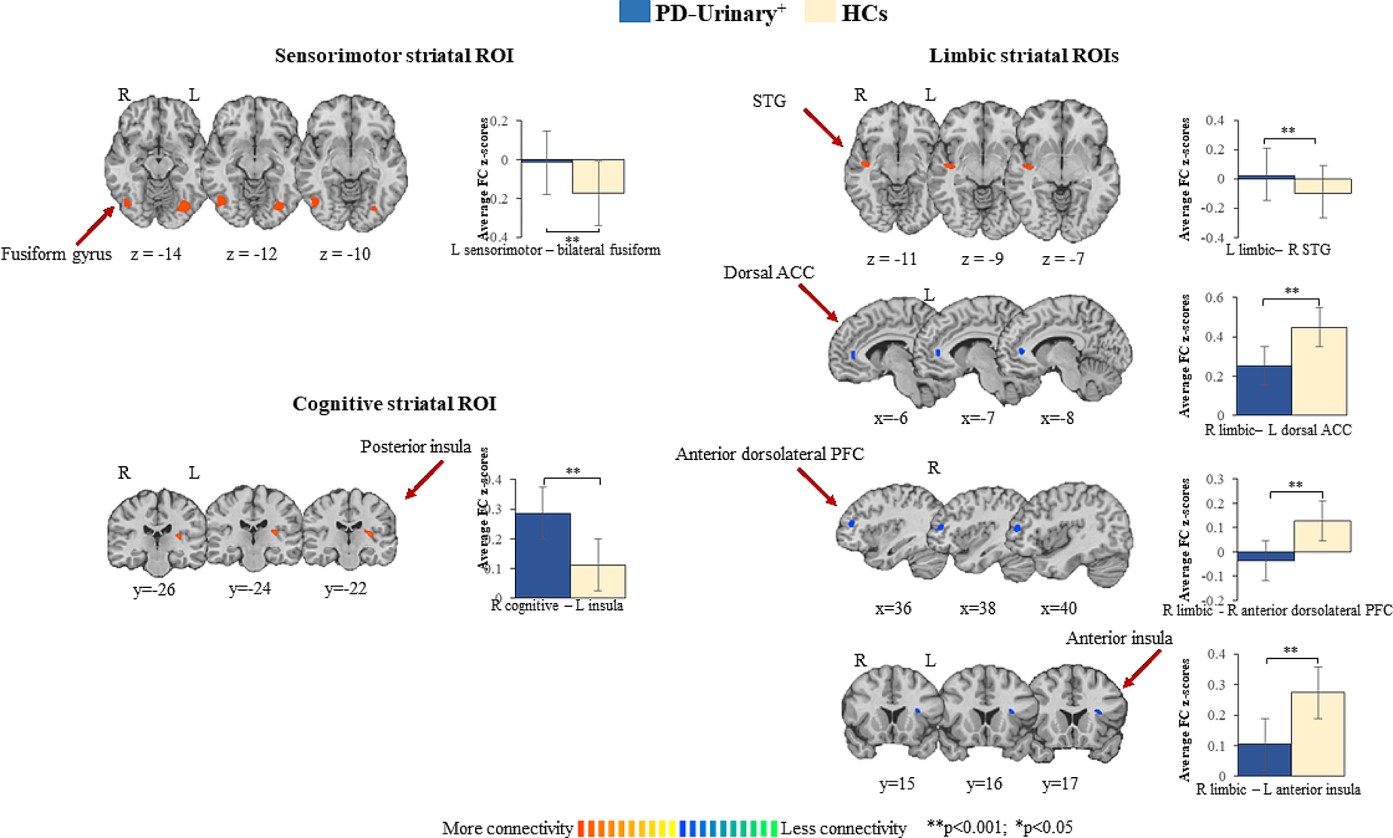
Striatal connectivity changes differentiating PD-urinary^+^ from HCs. Seed-based significant connectivity differences between PD-urinary^+^ and HCs and bar graphs of the average functional connectivity z-scores (for bilateral results, the mean of the two areas has been reported; please refer to Table 2 for full comparisons). Cold colors represent less, and hot colors represent more connectivity in PD-urinary^+^ relative to HCs (*p* < 0.05 cluster-level corrected). *Abbreviations*: ACC, anterior cingulate cortex; HCs, healthy controls; PD, Parkinson’s disease; PFC: prefrontal cortex; ROI: region of interest; STG: superior temporal gyrus

PD-urinary^+^ vs. controls (Fig. 2; Table 2).

Compared to HCs, PD-urinary^+^ patients showed increased FC between the right sensorimotor ROI and the bilateral fusiform gyri. Compared to HCs, PD-urinary^+^ patients showed increased FC between the left limbic ROI and the right superior temporal gyrus, and decreased FC between the right limbic ROI and the left anterior insula, the left dorsal ACC and the right anterior dorsolateral PFC. Compared to HCs, PD-urinary^+^ patients showed increased FC between the right cognitive ROI and the left posterior insula.

**Fig. 3:**
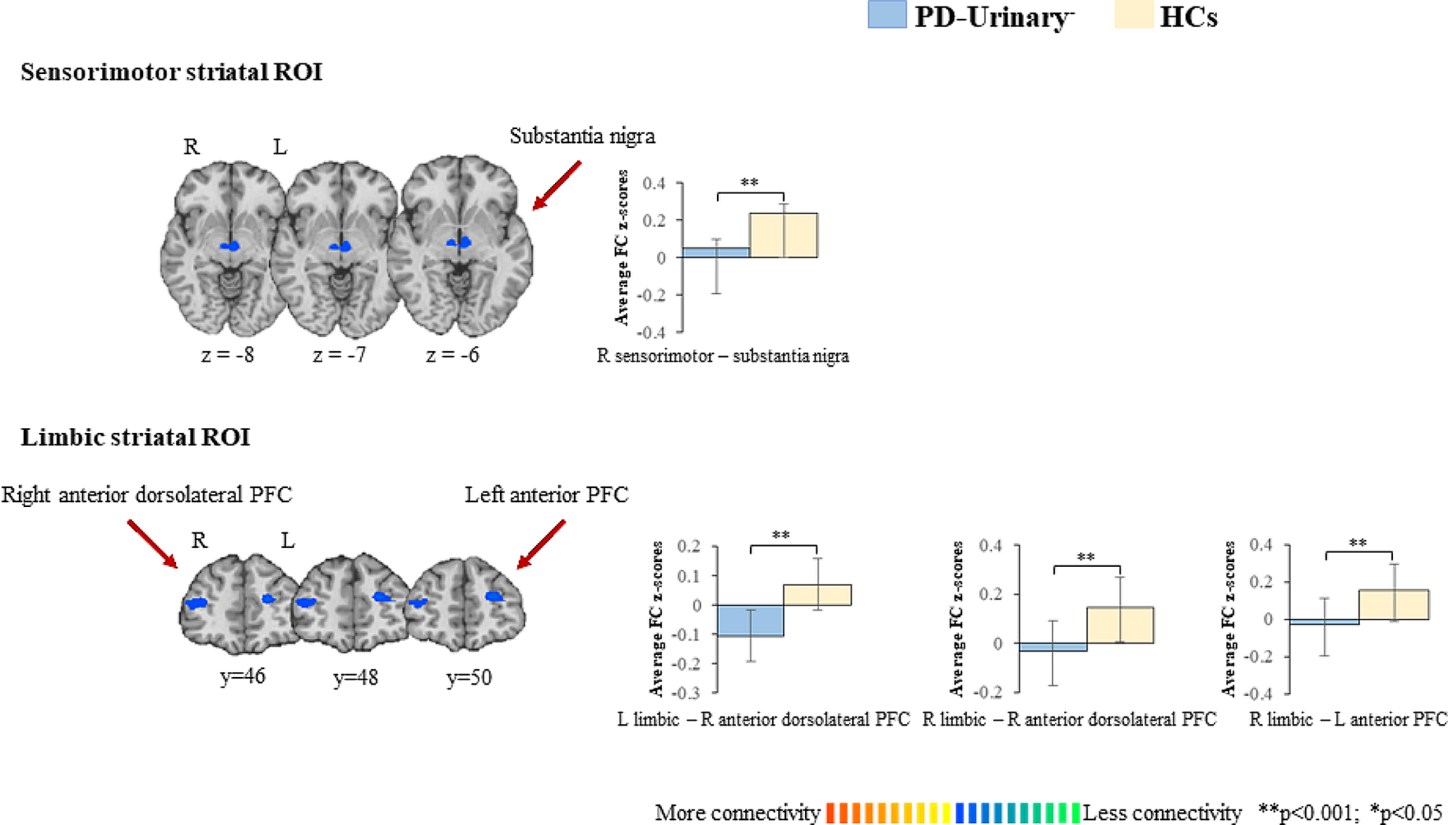
Striatal connectivity changes differentiating PD-urinary^−^ from HCs. Seed-based significant connectivity differences between PD-urinary^−^ and HCs, and bar graphs of the average functional connectivity z-scores (for bilateral results, the mean of the two areas has been reported; please refer to Table 2 for full comparisons). Cold colors represent less, and hot colors represent more connectivity in PD-urinary^−^ relative to HCs (*p* < 0.05 cluster-level corrected). Abbreviations: HCs, healthy controls; PD, Parkinson’s disease; PFC: prefrontal cortex; ROI: region of interest

PD-urinary^−^ vs. controls (Fig. 3; Table 2).

Compared to HCs, PD-urinary^−^ patients showed decreased FC between the right sensorimotor ROI and the bilateral substantia nigra. Compared to HCs, PD-urinary^−^ patients showed decreased FC between both the left and right limbic ROIs and the left anterior PFC and right anterior dorsolateral PFC.

**Table 2:** Summary of the significant functional connectivity differences between the study groups

Brain region (coordinates)	All PD mean ± SD	PD-urinary^+^ mean ± SD	PD-urinary^−^ mean ± SD	HCs mean ± SD	All PDvs HCs p-value	PD- urinary^+^ vs. HCs p-value	PD- urinary^−^ vs. HCs p-value	PD- urinary^+^ vs. PD-urinary^−^p-value
	N = 84	N = 45	N = 34	N = 38				
	*Left sensorimotor striatal ROI*							
Right PMA (x = 42; y=-13; z = 56)	-	0.15 ± 0.19	-0.00 ± 0.13	-	-	-	-	0.001
	*Right sensorimotor striatal ROI*							
Right fusiform gyrus (x = 39; y=-70; z=-14)	-0.0 ± 0.2	-	-	-0.2 ± 0.2	< 0.001	-	-	-
Substantia nigra (x=-6; y=-14; z=-7)	0.1 ± 0.2	-	-	0.2 ± 0.1	< 0.001	-	-	-
Substantia nigra (x = 5; y=-14; z=-7)	-	-	0.0 ± 0.2	0.2 ± 0.2	-	-	< 0.001	-
Left fusiform gyrus (x=-33; y=-76; z=-14)	-	0.0 ± 0.2	-	-0.1 ± 0.2	-	< 0.001	-	-
Right fusiform gyrus (x = 45; y=-67; z=-11)	-	0.0 ± 0.2	-	-0.2 ± 0.2	-	< 0.001	-	-
Right SMA (x = 6; y=-7; z = 52)	-	0.3 ± 0.1	0.1 ± 0.2	-	-	-	-	< 0.001
Right premotor area (x = 38; y=-13; z = 56)	-	0.2 ± 0.2	0.0 ± 0.2	-	-	-	-	< 0.001
Right angular gyrus (x = 33; -79; 34)	-	-0.2 ± 0.2	-0.0 ± 0.2	-	-	-	-	< 0.001
	*Left limbic striatal ROI*							
Left dorsal ACC (x=-13; y = 41; z = 9)	0.2 ± 0.2	-	-	0.4 ± 0.2	< 0.001	-	-	-
Right anterior dorsolateral PFC (x = 33; y = 44; z = 12)	-	-	-0.1 ± 0.1	0.2 ± 0.2	-	-	< 0.001	-
Right STG (x = 46; y=-11; z=-7)	-	0.1 ± 0.2	-	-0.1 ± 0.2	-	< 0.001	-	-
	*Right limbic striatal ROI*							
Right anterior dorsolateral PFC (x = 33; y = 44; z = 12)	-0.0 ± 0.2	-	-	0.1 ± 0.2	< 0.001	-	-	-
Left dorsal ACC (x=-15; y = 41; z = 9)	0.2 ± 0.2	-	-	0.4 ± 0.2	< 0.001	-	-	-
Left anterior insula (x=-30; y = 16; z = 14)	0.1 ± 0.2	-	-	0.2 ± 0.1	< 0.001	-	-	-
Left anterior PFC (x=-18; y = 47; z = 16)	-	-	-0.0 ± 0.1	0.2 ± 0.2	-	-	< 0.001	-
Right anterior dorsolateral PFC (x = 39; y = 50; z = 10)	-	-	-0.0 ± 0.2	0.1 ± 0.2	-	-	< 0.001	-
Right dorsolateral anterior PFC (x = 36; y = 44; z = 16)	-	-0.0 ± 0.2	-	0.1 ± 0.2	-	< 0.001	-	-
Left anterior insula (x=-27; y = 23; z = 16)	-	0.1 ± 0.2	-	0.3 ± 0.1	-	< 0.001	-	-
Left dorsal ACC (x=-9; y = 38; z = 7)	-	0.3 ± 0.2	-	0.4 ± 0.2	-	< 0.001	-	-
Left anterior PFC (x=-23; y = 50; z = 17)	-	0.0 ± 0.2	-0.1 ± 0.1	-				0.001
	*Left cognitive ROI*							
-	-	-	-	-	-	-	-	-
	*Right cognitive ROI*							
Left angular gyrus (x=-27; y=-64; z=-79)	-0.1 ± 0.2	-	-	-0.0 ± 0.1	< 0.001	-	-	-
Left posterior insula (x=-33; y=-21; z = 11)	0.2 ± 0.2	-	-	0.1 ± 0.2	< 0.001	-	-	-
Left posterior insula (x=-30; y=-25; z = 10)	-	0.2 ± 0.1	-	0.1 ± 0.2	-	< 0.001	-	-

P-values refer to pairwise T-test. A *p* < 0.002 (0.05/24) was considered statistically significant. Abbreviations: ROI, region of interest; ACC, anterior cingulate cortex; HCs, healthy controls; PD, Parkinson’s disease; PFC, prefrontal cortex; PMA: primary motor area; SMA, supplementary motor area; STG, superior temporal gyrus; SD, standard deviation

### Partial correlation analysis

No significant correlations were found between imaging data (i.e., FC z-scores) and NMSS – domain 7 scores in PD-urinary^+^.

### VBM results

VBM did not reveal statistically significant differences between all PD patients and HCs, as well as between the two PD subgroups and between each PD subgroup and HCs, across the whole brain, including the regions that were here used as seeds for, or resulted from the whole-brain analysis of, FC differences between PD-urinary^+^ and PD-urinary^−^ patients.

## Discussion

In this study, we investigated the possible association of urinary symptoms with the whole-brain FC pattern as emerging from distinct striatal subregions in a cohort of drug-naïve PD patients.

We found that the presence of urinary symptoms is associated with functional rearrangements within all the urinary-related central neural circuits.

The first circuit, encompassing the anterior PFC and the insula, is responsible for conscious and social control of bladder function and for awareness of visceral-motor/sensory functions. (Kavia et al. 2005)

The anterior PFC is crucial for planning complex motor and cognitive behaviors. Moreover, it has been proposed that this area, together with its connections with the limbic circuit, may lead to a voluntary control over the decision to void in a particular place/time (Ramnani and Owen 2004; Kavia and Mumtaz 2005), eventually inhibiting micturition in inappropriate situations. (Taylor et al. 2004; Kavia et al. 2005) We found an increased FC between the limbic striatal subregion and the anterior PFC in PD-urinary^+^ compared to PD-urinary^-^ patients. This may potentially be related to the presence of an abnormal response occurring in patients with urinary disturbances to delay the micturition.

We found an increased FC between the cognitive striatal ROI and the posterior insula in PD-urinary^+^ compared to HCs. The posterior insula is responsible for the cognitive elaboration, awareness, and sensorimotor/homeostatic processing (Uddin et al. 2017; Cera et al. 2020). This area has also been shown to be involved in bladder sensation and voluntary initiation/delay of micturition in patients with overactive bladder (Kavia and Mumtaz 2005; Uddin et al. 2017; Cera et al. 2020). A stronger recruitment of this area may occur in PD patients with urinary disturbances to sustain the cognitive effort needed to control the perception of fullness of the bladder. This speculation is in line with previous functional neuroimaging studies reporting an increased insular activation in conditions of awareness of sensation of thirst or bladder/rectum/stomach distention (Uddin et al. 2017).

Compared to controls, PD-urinary^+^ patients showed also a specific functional disruption between the limbic striatum and the anterior insula. It has been suggested that the two insular subregions are linked in a “posterior-to-anterior” way: the posterior region may elaborate the perception of stimuli that are consequently emotionally evaluated and integrated by the anterior insula. (Uddin et al. 2017; Cera et al. 2020)

Thus, a potential role of this region is more likely centered on the emotional control of appropriate voiding. Consequently, some studies assigned the anterior insula to the second neural circuit (Kitta et al. 2015).

The second urinary-related neural circuit is thought to include the dorsal ACC and the SMA, engaged in the elaboration of emotional and motor responses to voiding stimuli (Christopher et al. 2014; Kitta et al. 2015; Putcha et al. 2016) The ACC has been involved in regulation of interoceptive awareness upon bladder sensation. Moreover, it has been shown to be involved also in the control of the autonomic nervous system, and in the micturition process. (Kitta et al. 2015) We found a decreased FC between the dorsal ACC and the limbic striatum in PD-urinary^+^ patients compared to HCs, potentially leading to stimuli processing disturbances, including bladder filling. (Christopher et al. 2014; Putcha et al. 2016) This may ultimately lead to urinary frequency and urgency in PD patients. In line with our findings, the co-activation of the insula and the dorsal ACC has been proposed to regulate the integration of the limbic and autonomic responses. (Christopher et al. 2014; Kitta et al. 2015)

Compared to PD-urinary^−^, PD-urinary^+^ patients showed an increased FC between the sensorimotor striatal subregion and the SMA, premotor area and PMA. Together with the dorsal ACC, in healthy brains the SMA is deactivated during bladder filling, and this is considered as a back-up continence mechanism. (Griffiths 2015; de Groat et al. 2015) Thus, we might hypothesize that this functional pattern may be related to the re-programming of the motor output which may occur within the SMA and premotor area during bladder filling due to the perceived urinary frequency and urgency, while the PMA could be activated to recruit pelvic floor muscles to delay micturition. Accordingly, Di Gangi Herms and colleagues demonstrated that the PMA is activated in subjects with stress urinary incontinence after pelvic floor muscle training (Di Gangi Herms et al. 2006), suggesting a potential role for this area in driving increased pelvic floor contractions to delay micturition. A similar pattern has been found in PD patients with urinary dysfunction using PET imaging. (Kitta et al. 2015) Moreover, previous studies reported an increased activation of the SMA and PMA during voluntary pelvic floor muscle contractions in full-bladder conditions, and/or during the perception of increased urge to void in healthy subjects, (Blok et al. 1997; Kuhtz-Buschbeck et al. 2005, 2007; Zhang et al. 2005) in subjects with stress urinary incontinence, (Di Gangi Herms et al. 2006) and in patients with overactive bladder. (Kitta et al. 2015) In this framework, previous small-scale studies have demonstrated the feasibility and effectiveness of noninvasive brain stimulation, such as repetitive transcranial magnetic stimulation (rTMS) targeted at inhibiting the SMA in patients with PD and urinary disturbances (Brusa et al. 2009).

We found a decreased FC between the sensorimotor striatal ROI and the right angular gyrus in PD-urinary^+^ compared to PD-urinary^-^ patients. The inferior parietal lobule is implicated in the selection and monitoring of motor sequences. (Deiber et al. 1991; Grafton et al. 1992; Wu et al. 2009) Previous studies showed an increased activity of this region, together with the SMA and PMA, during voluntary pelvic floor muscle contractions in healthy subjects, suggesting a potential role of the frontoparietal areas in the sensorimotor integration and timing of voiding. (Blok et al. 1997; Kuhtz-Buschbeck et al. 2007) Thus, our findings suggest that a potential breakdown of these regulation processes may occur in PD patients with urinary symptoms.

Finally, the third urinary-related neural circuit, involving temporal area and the basal ganglia, is thought to be relevant to perform a subconscious monitoring of bladder events. (Griffiths 2015; de Groat et al. 2015)

We found an increased FC between the limbic striatal ROI and the temporal gyrus in PD-urinary^+^ patients compared to HCs. Previous studies demonstrated a role of this cortical region in the allocentric spatial processing in healthy subjects, meaning that it is involved in the mnemonic representation of episodic scenes and relations between items in local environments. (Galati et al. 2000; Neggers et al. 2006; Aminoff et al. 2013) Patients with urinary dysfunctions are more prone to memorize the local space to detect the easiest way to reach the bathroom (Tish and Geerling 2020). Thus, we might speculate that in PD-urinary^+^ patients there may be a hyper-recruitment of the temporal gyrus and its connections to the limbic striatum. The presence of a compensatory functional rearrangement within these areas is in line with previous studies showing that early PD patients may rely on spared cortical regions to maintain good global performances in the initial stages of the disease. (De Micco et al. 2021a)

Finally, PD-urinary^+^ patients showed increased FC between the sensorimotor striatal ROI and the fusiform gyri compared to HCs. Evidence supporting the role of the occipital cortex in urinary dysfunction in both healthy subjects and PD patients are sparse. It has been suggested that the occipital cortex may be involved in the bladder sensation of increasing levels of urine in healthy subjects. (Mehnert et al. 2008; Griffiths 2015) Moreover, an amplified activation of occipital areas has been found during a marked bladder volume increase in subjects with poor control activation. (Griffiths 2015) Having that in mind, we might speculate that the increased FC between the sensorimotor ROI and the fusiform gyri that we found in PD-urinary^+^ patients may be related to the attempt to compensate for the poor control activation of the bladder.

Our study has some limitations. We applied only the NMSS 7 to detect the presence of urinary symptoms, and no proper urological/urodynamic assessment was performed. This, it is not possible to completely rule out the presence of other or concomitant etiologies other than PD for the urinary symptoms complained by patients. However, it should be considered that PD patients with history of urologic disorders (i.e. indwelling catheter, renal dialysis, cardiac failure requiring diuretics, urinary tract infection, prostate or bladder cancer, uncontrolled bladder outlet obstruction, pelvic organ prolapse, and previous urogynecologic surgery) were excluded from this study, to reduce the potential bias emerging from the presence of peripheric causes of urinary symptoms. Moreover, by using the total score of the NMSS domain 7, we considered urinary urgency and frequency together, and not separately. Thus, it is not possible to ascertain whether our striatal neural correlates in PD patients were related only to one of these symptoms or both. However, our study design is consistent with previous studies focusing on urinary disturbances in PD patients. (Hou et al. 2016; Picillo et al. 2017)

We found that PD-urinary + patients reported also more severe gastrointestinal symptoms compared to PD-urinary-. This is not surprising, as urinary and gastrointestinal disturbances are highly correlated in the general population and in PD as well.(Picillo et al. 2017) Nevertheless, a second-level analysis showed that FC differences between PD-urinary^+^ and PD-urinary^−^ were still strongly significant even after correcting for other NMSS items scores, likely suggesting that the presence of gastrointestinal symptoms did not influence our results.

We did not find any significant correlations between the MRI findings (i.e., FC z- scores) and NMSS – domain 7 scores in PD-urinary^+^. This may be potentially due to the small sample size. Moreover, it should be noted that we enrolled early PD patients with mild to moderate urinary symptoms, reporting similar scores at the NMSS – domain 7 (i.e., low standard deviation), and this may also lead to the absence of significant correlations with imaging data.

Finally, we acknowledge that subjects with urinary symptoms may have felt the need to inhibit the micturition during the scanning time. As this study was planned to be acquired in resting-state condition, subjects were requested to urinate immediately before entering in the MRI and to stop the image acquisition in case they felt any need to urinate. All PD patients (and controls) enrolled in this study were able to undergo a 45-minutes MRI examination without reporting urinary urgency during the scanning time.

## Conclusions

In conclusion, we showed that specific FC changes may be found in PD patients with urinary symptoms all over the forebrain neural circuits that are physiologically involved in this complex function: (i) increased FC between striatal regions and insula/anterior dorsolateral PFC, aiming at inhibiting the urinary urge while in social context and to perform the cognitively control over the perception of bladder emptiness/fullness; (ii) increased FC between striatal regions and motor and premotor/supplementary motor areas as an attempt to recruit the pelvic floor muscles and overcome urinary frequency and urgency to delay micturition; (iii) decreased FC between striatal regions and parietal, insular and cingulate cortices as an early biomarker for altered autonomic and behavioral sensorimotor integration of the timing of voiding; (iv) increased FC between striatal regions and temporo-occipital areas as a compensatory mechanism for altered sensorimotor control. These results may provide new insights into the neural correlates of urinary symptoms in PD patients and may potentially help clinicians to design more tailored neuromodulation protocols targeting the neural circuitry that are crucially involved in the micturition cycle to restore or reroute autonomic and sensorimotor activity between the brain and bladder.

### Electronic supplementary material

Below is the link to the electronic supplementary material.

Supplementary Material 1

Supplementary Figure 1

## Data Availability

The data that support the findings of this study are available from the corresponding author upon reasonable request.
