# Supplementary material for: Altered domain-specific striatal functional connectivity in patients with Parkinson’s disease and urinary symptoms
Source: J Neural Transm (Vienna). 2024 Apr 25;131(8):917–29. doi: 10.1007/s00702-024-02776-0 (PMC11343795; doi:10.1007/s00702-024-02776-0)
Supplement: Supplementary file 1 — Supplementary Material 1 [file 702_2024_2776_MOESM1_ESM.docx]

**Supplementary materials**

*Clinical Evaluation – Neuropsychological and behavioral symptoms*

Global cognitive functioning was assessed by the Montreal Cognitive Assessment (MoCA) (Nasreddine et al. 2005; Santangelo et al. 2015) and Mini-Mental State Examination (MMSE) (Folstein et al. 1975) in patients and healthy controls, respectively. Moreover, PD patients performed a neuropsychological battery that included several tests and rescored MoCA items to explore the following cognitive domains: attention and working memory

(Trail Making Test A (Giovagnoli et al. 1996), forward digitspan (Monaco et al. 2013), and backward digit span item of the MoCA graded using a modified 0 to 2 rating scale (Fengler et al. 2016)); memory [Rey Auditory Verbal Learning Test (delayed recall (Carlesimo et al. 1996)), and prose recall test (Novelli et al. 1986); executive functions [by means of a letter fluency task (Carlesimo et al. 1996), Modified Card Sorting Test (number of achieved categories)] (Caffarra et al. 2004); visuospatial abilities [by means of the Rey−Osterrieth Complex Figure Test (copy) (Caffarra et al. 2004), and cube copy item of the MoCA, graded using a modified 0 to 3 rating scale (Fengler et al. 2016); and language (naming and sentence repetition items of the MoCA, graded using modified 0 to 1 and 0 to 3 rating scales, respectively) (Fengler et al. 2016). Performance on the individual neuropsychological test was transformed into z-scores. Subsequently, a composite summary index for each cognitive domain was derived from the corresponding averages of the respective neuropsychological tests (ie, attention and working memory z-score, memory z-score, executive functions z-score, visuospatial abilities z-score, and language z-score).

Depressive symptoms were assessed with the Beck Depression Index (BDI-II) (Leentjens et al. 2000), anxiety symptoms were assessed with the Parkinson Anxiety Scale (PAS) (Leentjens et al. 2014), fatigue severity was assessed by the Parkinson Fatigue scale (PFS) (Friedman et al. 2010), apathy with the Apathy Evaluation Scale (AES) (Marin et al. 1991).

*VBM analysis*

Data were processed and examined using SPM12 software (Wellcome Trust Centre for Neuroimaging, London, UK; http://www.fil.ion.ucl.ac.uk/spm) and Data Processing & Analysis of Brain Imaging (DPABI, Yan et al. 2016, http://rfmri.org/DPABI). Each structural image was segmented into gray matter, white matter and cerebrospinal fluid using a fully automated algorithm within DPABI and subsequently transformed to Montreal Neurological Institute (MNI) space using DARTEL-normalization. Next, the normalized gray matter images were modulated (via scaling to the Jacobian determinants of the deformations) and smoothed (FWHM = 8 mm) for statistical analyses. The GM volume maps were statistically analyzed using the general linear model based on Gaussian random field theory. Statistical modeling consisted of a full factorial design with group as factor of interest and age, sex and total intracranial volume (TIV) as covariates of no-interest. Statistical inference on the main effects of group (contrast: All PD vs. HC) was performed at the voxel level over the whole brain with a family-wise error (FWE) correction for multiple comparisons (p<0.05). Moreover, from the same model, a whole-brain voxel-based analysis of the effect of the presence of urinary symptoms was performed on the restricted group of PD patients (contrast: PD-urinary^+^ vs. PD-urinary^-^) to verify whether any GM volume differences could be also detected across specific regions that were either used as seeds for, or resulted from the whole-brain analysis of, FC differences between PD-urinary^+^ and PD-urinary^-^ patients.

**References**

Caffarra P, Vezzadini G, Dieci F, et al (2004) Modified Card Sorting Test: normative data. J Clin Exp Neuropsychol 26:246–250. https://doi.org/10.1076/JCEN.26.2.246.28087

Carlesimo GA, Caltagirone C, Gainotti G, et al (1996) The Mental Deterioration Battery: normative data, diagnostic reliability and qualitative analyses of cognitive impairment. The Group for the Standardization of the Mental Deterioration Battery. Eur Neurol 36:378–384. https://doi.org/10.1159/000117297

Fengler S, Kessler J, Timmermann L, et al (2016) Screening for Cognitive Impairment in Parkinson’s Disease: Improving the  Diagnostic Utility of the MoCA through Subtest Weighting. PLoS One 11:e0159318. https://doi.org/10.1371/journal.pone.0159318

Folstein MF, Folstein SE, McHugh PR (1975) “Mini-mental state”. A practical method for grading the cognitive state of patients for the clinician. J Psychiatr Res 12:189–198. https://doi.org/10.1016/0022-3956(75)90026-6

Friedman JH, Alves G, Hagell P, et al (2010) Fatigue rating scales critique and recommendations by the Movement Disorders  Society task force on rating scales for Parkinson’s disease. Mov Disord 25:805–822. https://doi.org/10.1002/mds.22989

Giovagnoli AR, Del Pesce M, Mascheroni S, et al (1996) Trail making test: normative values from 287 normal adult controls. Ital J Neurol Sci 17:305–309. https://doi.org/10.1007/BF01997792

Leentjens AF, Verhey FR, Luijckx GJ, Troost J (2000) The validity of the Beck Depression Inventory as a screening and diagnostic  instrument for depression in patients with Parkinson’s disease. Mov Disord 15:1221–1224. https://doi.org/10.1002/1531-8257(200011)15:6<1221::aid-mds1024>3.0.co;2-h

Leentjens AFG, Dujardin K, Pontone GM, et al (2014) The Parkinson Anxiety Scale (PAS): development and validation of a new anxiety scale. Mov Disord 29:1035–1043. https://doi.org/10.1002/MDS.25919

Marin RS, Biedrzycki RC, Firinciogullari S (1991) Reliability and validity of the Apathy Evaluation Scale. Psychiatry Res 38:143–162. https://doi.org/10.1016/0165-1781(91)90040-v

Monaco M, Costa A, Caltagirone C, Carlesimo GA (2013) Forward and backward span for verbal and visuo-spatial data: standardization and normative data from an Italian adult population. Neurol Sci 34:749–754. https://doi.org/10.1007/S10072-012-1130-X

Nasreddine ZS, Phillips NA, Bédirian V, et al (2005) The Montreal Cognitive Assessment, MoCA: a brief screening tool for mild cognitive impairment. J Am Geriatr Soc 53:695–699. https://doi.org/10.1111/J.1532-5415.2005.53221.X

Novelli G, Papagno C, Capitani E, et al (1986) Tre test clinici di memoria verbale a lungo termine: Taratura su soggetti normali. [Three clinical tests for the assessment of verbal long-term memory function: Norms from 320 normal subjects.]. Arch Psicol Neurol Psichiatr 47:278–296

Santangelo G, Siciliano M, Pedone R, et al (2015) Normative data for the Montreal Cognitive Assessment in an Italian population sample. Neurol Sci 36:585–591. https://doi.org/10.1007/S10072-014-1995-Y

**Supplementary results**

**Table S1** Univariate analysis of variance between the individual functional connectivity z-scores of PD-urinary+ and PD-Urinary- using the other NMSS items scores as covariates.

| **Brain region (coordinates)** | **PD-urinary^+^ mean ± SD** | **PD-urinary^-^ mean ± SD** | **Adj-p value** |
| --- | --- | --- | --- |
|  | **N=45** | **N=34** |  |
| *Left sensorimotor striatal ROI* | | | |
| Right PMA (x=42; y=-13; z=56) | 0.15±0.19 | -0.00±0.13 | 0.006 |
| *Right sensorimotor striatal ROI* | | | |
| Right SMA (x=6; y=-7; z=52) | 0.3±0.1 | 0.1±0.2 | 0.003 |
| Right premotor area (x=38; y=-13; z=56) | 0.2±0.2 | 0.0±0.2 | 0.004 |
| Right angular gyrus (x=33; -79; 34) | -0.2±0.2 | -0.0±0.2 | 0.003 |
| *Right limbic striatal ROI* | | | |
| Left anterior PFC (x=-23; y=50; z=17) | 0.0±0.2 | -0.1±0.1 | 0.006 |

P-values refer to pairwise T-test. Analyses were Bonferroni-corrected for multiple comparisons. Covariates: NMSS-1, Cardiovascular; NMSS-2, Sleep/fatigue; NMSS-3, Mood/apathy; NMSS-4, Perceptual problems; NMSS-5, Attention/Memory; NMSS-6, Gastrointestinal; NMSS-8, Sexual function; NMSS-9, Miscellaneus, Use of medications to omprove urinary disturbances (yes/no). Abbreviations: ROI, region of interest; PD, Parkinson’s disease; PFC, prefrontal cortex; PMA: primary motor area; SMA, supplementary motor area; SD, standard deviation.

**Figure legend**

**Figure S1.** Striatal connectivity changes differentiating all PD patients from HCs. Seed-based significant connectivity differences between all PD patients and HCs, and bar graphs of the average functional connectivity z-scores (for bilateral results, the mean of the two areas has been reported; please refer to Supplementary Table 2 for full comparisons). Cold colors represent less, and hot colors represent more connectivity in all PD patients relative to HCs (p<0.05 cluster-level corrected). Abbreviations: ACC, anterior cingulate cortex; HCs, healthy controls; PD, Parkinson’s disease; PFC: prefrontal cortex; ROI: region of interest; STG: superior temporal gyrus.
