# Supplementary figures and images for: Altered domain-specific striatal functional connectivity in patients with Parkinson’s disease and urinary symptoms
Source: J Neural Transm (Vienna). 2024 Apr 25;131(8):917–29. doi: 10.1007/s00702-024-02776-0 (PMC11343795; doi:10.1007/s00702-024-02776-0)

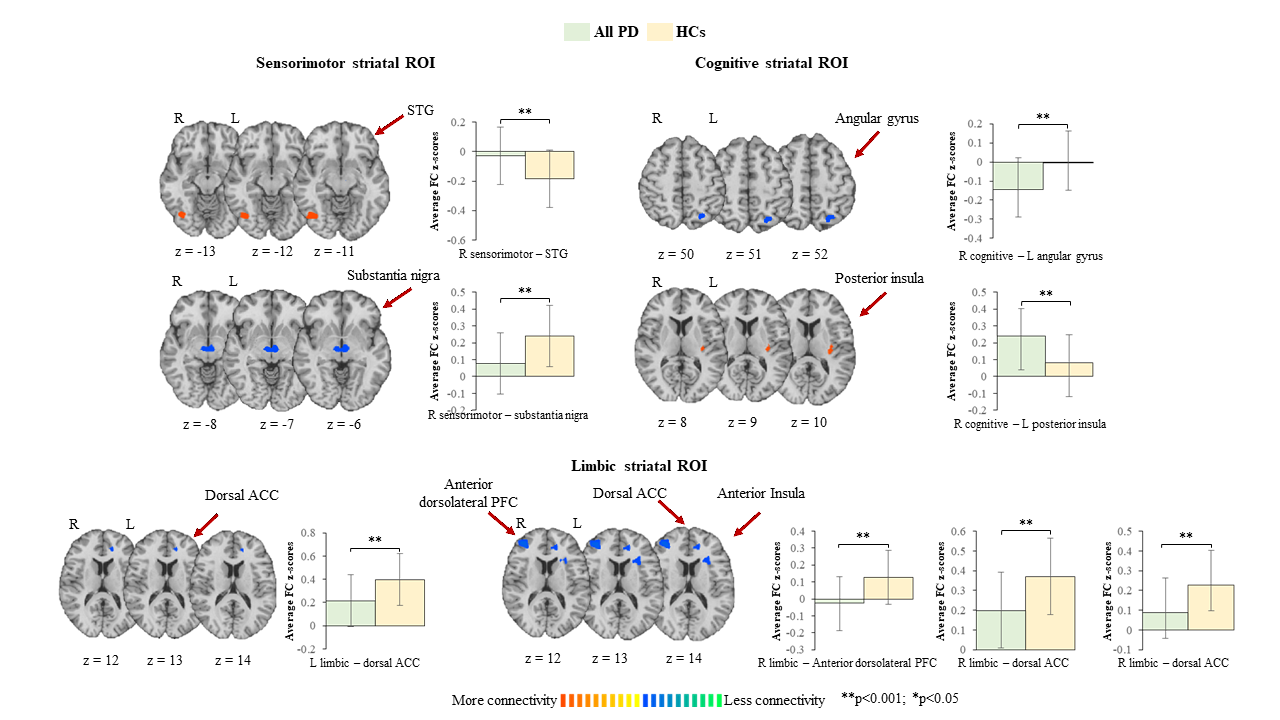

Supplement: Supplementary file 2 — Supplementary Figure 1 [file 702_2024_2776_MOESM2_ESM.tiff]
